# Microbiological Trends and Antibiotic Susceptibility Patterns in Patients with Periprosthetic Joint Infection of the Hip or Knee over 6 Years

**DOI:** 10.3390/antibiotics11091244

**Published:** 2022-09-13

**Authors:** Frank Sebastian Fröschen, Thomas Martin Randau, Alexander Franz, Ernst Molitor, Achim Hoerauf, Gunnar Thorben Rembert Hischebeth

**Affiliations:** 1 Department of Orthopaedics and Trauma Surgery, University Hospital Bonn, 53127 Bonn, Germany; 2 Institute of Medical Microbiology, Immunology and Parasitology, University Hospital Bonn, 53127 Bonn, Germany

**Keywords:** hip, knee, periprosthetic joint infection, antimicrobial resistance, microorganism, trend

## Abstract

We sought to analyze trends of the causative pathogens and their antibiotic susceptibility patterns in patients with periprosthetic joint infections (PJI) of the hip and knee to get better insights and improve treatment. Retrospective evaluation of all consecutive patients with microbiological detection of a causative pathogen at a tertiary endoprothetic referral center between January 2016 and December 2021 in Germany was performed. Overall, 612 different microorganisms could be detected in 493 patients (hip: *n* = 293; knee: *n* = 200). Evaluation did not show a change in the relative abundance of pathogens detected, with coagulase-negative staphylococci (*n* = 275; 44.9%) found frequently, followed by *S. aureus* (*n* = 86; 14.1%), *Enterococcus* species (*n* = 57; 9.3%), *Streptococcus* species (*n* = 48; 7.8%), and Gram-negative bacteria (*n* = 80; 13.1%). Evaluation of the antibiotic susceptibilities showed increasing rates of oxacillin-resistant coagulase-negative staphylococci (60.4%; 46.8–76.7%) and piperacillin-tazobactam-resistant Gram-negative bacteria (26.5%; 0–57.1%), although statistically not significant. Resistance of Gram-positive bacteria to vancomycin (<1%) and Gram-negative microorganisms to meropenem (1.25%) remained an exception. In summary, coagulase-negative staphylococci, as the most frequent pathogen, displayed a continuously high rate of oxacillin resistance. For the highest antimicrobial coverage in the case of an empiric therapy/unknown pathogen, vancomycin might be chosen. Level of evidence: IV.

## 1. Introduction

The best possible treatment of a periprosthetic joint infection (PJI) after total hip arthroplasty (THA) or total knee arthroplasty (TKA) has become a major focus of orthopaedic research [1,2,3]. An essential part of a successful treatment with eradication of infection is knowledge of the causative pathogens and their antibiotic susceptibility patterns, as reinfection still remains a major problem after reimplantation of THA and TKA [2,4,5,6,7].

Against this background, it is decisive to remember that reinfection might occur delayed, as Garvin et al. outlined, and affects all aspects of the patient’s life, presenting a severe psychological burden for the patient [8,9]. However, the prevalence of causative pathogens and their antibiotic resistances may vary [10]. In addition, identification of the causative pathogen might not be possible in all cases, especially for those patients with culture-negative PJI knowledge of epidemiological data, and antibiotic susceptibilities of the causative pathogens might be helpful to prevent recurrence of PJI. Here, Sebastian et al. described in this context differences in the microbiological profiles of patients with PJI between Sweden and Lithuania, with a higher rate of methicillin-resistant *S. aureus* in Lithuania than in Sweden, outlining the need for further data for better comparison [11].

Overall, several studies have tried to analyze microbiological profiles of patients with PJI of the hip and the knee with inconsistent results [12,13,14]. Although most describe Gram-positive cocci and coagulase-negative staphylococci as the most frequently detected pathogens in patients with PJI, the evaluation of antibiotic susceptibility patterns and changes of detection rates of causative pathogens remains a topic of ongoing debate [12,15]. Overall, only scant data exist regarding the evaluation of causative pathogens over time. Hu et al. and Bjerke-Kroll et al. detected an increase in methicillin-resistant staphylococci, raising the question of clinical relevance [14,15]. Nevertheless, both reported different detection rates of the causative pathogens, outlining the need for further studies to evaluate changes in antibiotic susceptibility patterns over the course of time [13,14]. The presented data may, therefore, be helpful to identify the best possible initial empiric antibiotic therapy until identification of the causative pathogen, or in patients with culture-negative PJI.

The purpose of this study was to analyze the causative pathogens detected over a period of six years in patients with PJI of the hip or knee. We aimed to assess if there is a change in the frequency of different detected pathogens over the course of time. Furthermore, we wanted to evaluate whether antibiotic susceptibilities against the most frequently detected pathogens have changed within the analyzed six-year period.

## 2. Results

Over a study period of six years, 493 patients with PJI of the hip (*n* = 293) or knee joint (*n* = 200) and microbiological detection of a causative pathogen could be included. Demographic data are available in Table 1.

The majority of the patients had a chronic late onset PJI (*n* = 310), followed by patients with an acute early onset (*n* = 90) or acute late onset (*n* = 90) PJI. Of note, 399 patients (81%) had a monomicrobial PJI, while 94 patients (19%) had a polymicrobial PJI. In 76 cases (15.4%), two pathogens could be detected, while in 11 cases (2.2%), detection of three pathogens was possible, and in 7 cases (1.4%), 4 different pathogens have been detected. In patients with PJI of the hip, debridement, antibiotics, implant retention (DAIR) was performed in 108 cases (PJI of the knee: *n* = 88), while explantation of the inlying implant was performed in 185 cases (PJI of the knee: *n* = 112). Mean surgery time was 156 ± 71 min (hip: 162 ± 74 min; knee: 148 ± 66 min). The most common comorbidities in patients with PJI were hypertension (88%), diabetes mellitus (35%) and smoking (29%). Patients with an acute late onset PJI displayed on average the highest serum C-reactive protein levels (131.65 ± 109.04 mg/L), while patients with a chronic late onset PJI showed the lowest serum C-reactive protein levels (53.56 ± 79.62 mg/L).

The distribution of the pathogens detected over the analyzed six-year period is displayed in Table 2.

Overall, 612 different microorganisms have been identified; 83.6% of all organisms detected were Gram-positive bacteria, followed by Gram-negative bacteria (13.1%) and fungi (2.4%). The overall most frequently detected pathogens were coagulase-negative staphylococci (*n* = 275; 44.9%), *S. aureus* (*n* = 86; 14.1%), *Enterococcus* species (*n* = 57; 9.3%) and *Streptococcus* species (*n* = 48; 7.8%). The detected Gram-negative organisms showed an inhomogeneous distribution. In total, 13 different species could be detected, with *E. coli* (3.4%) and *P. mirabilis* (2.4%) being the most common ones.

**Figure 1:**
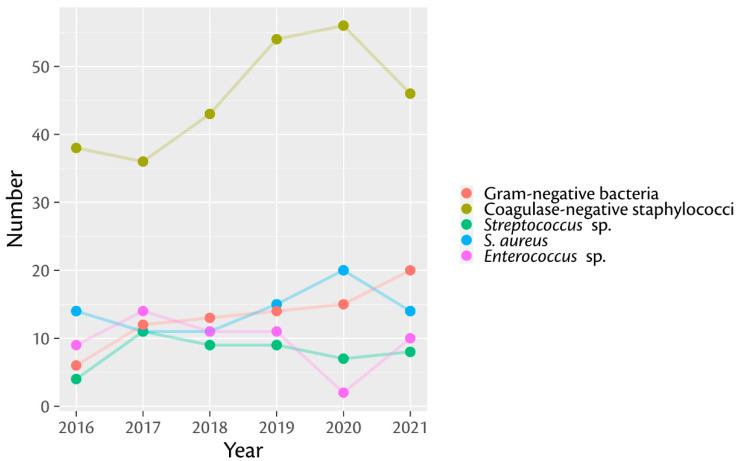
Annual detection rates of the five most common pathogens from 2016 to 2021 (vertical axis: number of detected pathogens; horizontal axis: years; sp.: species).

Analysis of the detected pathogens per year displayed a steady state (Figure 1), with coagulase-negative staphylococci (average: 45.1%; range: 36.7–50.6%) being the most common group in all analyzed years, followed by *S. aureus* (average 14.2%; range: 11.2–20%), *Enterococcus* species (average 9.6%; range: 1.6–14.2%) and Gram-negative bacteria (average 7.8%; range: 8–13.54%). In contrast, detection rates of Gram-negative bacteria revealed *E. coli* as being the most frequently detected pathogen except in 2018 and 2019, where *P. mirabilis* was the most frequently detected Gram-negative organism. Detection of fungi was seen in all years studied, but only in a minority of all PJIs (15 of 493 cases).

Statistical analysis for coagulase-negative staphylococci (*p* = 0.52)*, S. aureus* (*p* = 0.85) and Gram-negative bacteria (*p* = 0.31) did not show a significant change in the detection rates during the analyzed period. For enterococci (*p* = 0.012), an increase could be detected which did not reach statistical significance after performing the Bonferroni correction for adjusting the α-value.

Table 3 displays the results of the antibiotic susceptibility testing for coagulase-negative staphylococci, *S. aureus*, *Enterococcus* species, *Streptococcus* species and Gram-negative bacteria. In detail, *S. aureus* displayed an average oxacillin resistance rate of 7.4% (range: 0 to 21.4% per year) and an average rifampicin resistance rate of 7.8% (range: 0 to 21.4% per year). No *S. aureus* isolate tested within the six-year period displayed a vancomycin resistance. In contrast, coagulase-negative staphylococci showed on average an oxacillin resistance rate of 60.4% (range: 46.8–76.7%) and an average rifampicin resistance rate of 24.4% per year (range: 12.5–34.55%), while resistance against vancomycin could only be detected on average in 0.77% of isolated strains per year (range: 0–2.7%).

For enterococci, only *E. faecium* displayed in all isolates (*n* = 11) a resistance to ampicillin and in two isolates an additional resistance against vancomycin. All *E. faecalis* isolates (*n* = 46) proved to be susceptible to ampicillin and vancomycin. Gram-negative organisms showed on average an overall resistance-rate to piperacillin/tazobactam of 26.5% (range: 0–57.1% per year; ciprofloxacin: 17.3%, range: 0–35.71%; meropenem: 1%; range: 0–7.1%).

Statistical evaluation did not show a significant change in resistance rates after Bonferroni correction for adjusting the α-value for *S. aureus* (oxacillin: *p* = 0.211, rifampicin *p* = 0.035), coagulase-negative staphylococci (oxacillin: *p* = 0.016, rifampicin: *p* = 0.76), enterococci (ampicillin: *p* = 0.76; vancomycin: *p* = 0.77) or Gram-negative bacteria (ciprofloxacin: *p* = 0.140, piperacillin/tazobactam: *p* = 0.021; meropenem: *p* = 0.419).

In summary, we could detect an increase in oxacillin resistance of coagulase-negative staphylococci and piperacillin/tazobactam resistance of Gram-negative bacteria, which did not reach statistical significance.

To further analyse the most common pathogen (coagulase-negative staphylococci), a subgroup analysis was performed. In 70.5%, coagulase-negative staphylococci were detected in patients with a chronic late onset PJI (acute early onset: 17.8%; persisting early onset: 0.4%; acute late onset: 11.3%). Detection of coagulase-negative staphylococci was possible on average in 2.6 ± 2 intraoperative specimens (chronic late onset: 2.5 ± 1.9). Antibiotic susceptibility testing revealed an oxacillin resistance of coagulase-negative staphylococci in the majority of the analysed strains in all subgroups (acute early onset PJI: 68.1% of 47 isolates; acute late onset: 53.3% of 30 isolates; chronic late onset: 59.9% of 191 isolates), except in the subgroup persisting in early onset infection (detection of only one oxacillin-susceptible isolate). There was no significant difference in oxacillin resistance rates of coagulase-negative staphylococci in patients with PJI under consideration of the subgroup (*p* > 0.05).

## 3. Discussion

Although PJI is a rare complication after total joint arthroplasty with an overall incidence of 0.3–2%, it remains a challenging situation for the orthopaedic surgeon [2,4,16]. For successful treatment, not only is identification of the causative pathogen essential, but also knowledge of its antibiotic susceptibility. With this study, we present a large cohort of patients with PJI derived from a tertiary endoprosthetic referral center in Germany. Those data might therefore support orthopaedic surgeons during treatment of patients with PJI.

Although previous studies have shown that Gram-positive cocci are the most frequently detected pathogens in patients with PJI, the literature is not consistent regarding the role of *S. aureus* and coagulase-negative staphylococci [3,12]. While Tsai et al. identified *S. aureus* as the most common pathogen with a detection rate of 26%, more recent studies by Hu et al. and Stevoska et al. identified coagulase-negative staphylococci with detection rates of 38.1 to 56.6% as more common [12,15,17]. In accordance with the more recent studies, our most frequently detected pathogens are coagulase-negative staphylococci (44.9%), followed by *S. aureus* (14.1%), Gram-negative bacteria (13.1%) and *Enterococcus* species (9.3%). The detection rates of Gram-negative bacteria are comparable to previously reported rates ranging from 5 to 20%, while the detection rate of enterococci in our cohort was higher than reported in the literature, with described rates ranging from 3.8 to 5.2% [14,15].

An additional frequently discussed aspect of PJI is a possible difference of the causative pathogens in patients with PJI of the hip and the knee. While Bjerke-Kroll et al. could not detect a difference in the identified species in patients with PJI of the hip or knee, Stevoska et al. described a significantly higher rate of Gram-negative bacteria in patients with PJI of the hip [14,17]. As our previous studies did not detect an overall difference in causative pathogens between patients with PJI of the hip and the knee, we did not evaluate this topic [3].

To evaluate changes in the detection rates of causative pathogens in patients with PJI, we performed a subgroup analysis of the causative pathogens per year. Interestingly, we could not detect a change in the relative abundancy of the different taxa. Over the analysed six-year period, coagulase-negative staphylococci remained the most frequently detected pathogens, followed by *S. aureus*, Gram-negative bacteria, *Enterococcus* species and *Streptococcus* species. This remains a relevant aspect as changes over the course of time remain a topic of ongoing debate, while in general only scant data exist. In this context, Stefansdottir et al. reported an increase of PJI with detection of coagulase-negative staphylococci from 1986 to 2000 in Sweden, while describing a decrease in the number of infections caused by *S. aureus* and enterococci [18]. Results that are not consistent with data of patients with PJI from 2006 to 2015 in China were published by Hu et al., who described a slight decrease in the proportions of *S. aureus*, coagulase-negative staphylococci and enterococci as causative pathogens for PJI [15]. For interpretation of the results mentioned, it is essential to know that Hu et al. analysed in total 231 cases over nine years (93 cases: 2006–2010; 138 cases 2011–2015), while Stefansdottir et al. analysed 426 cases over 14 years [15,18]. Both authors state that further studies are necessary to identify and evaluate trends of causative pathogens in PJI. In contrast to these studies, we could not detect a change in the detection rate of causative pathogens during the analysed six-year period. Here, it must be considered that our analysed cohort with 493 included patients remains one of the largest analysed to date. Our data show a consistent and stable distribution of causative pathogens in patients with PJI. We agree that further studies are needed to optimize treatment and support the interdisciplinary team in successful treatment of PJI.

For optimization of the treatment of patients with PJI, not only the knowledge of the causative pathogen, but also the results of the antibiotic susceptibility testing might be helpful. A targeted antibiotic therapy is essential for treatment success. In the case of an unknown pathogen, the orthopaedic surgeon must rely on recommendations. To date, empirical antibiotic therapy with ampicillin-sulbactam or amoxicillin-clavulanic acid is often recommended as a first-line therapy [19]. As recommendations differ between countries, only the knowledge of the local microbiological spectrum might allow the best possible choice [2,3]. Our main findings for Gram-positive cocci are that coagulase-negative staphylococci display in the majority of the isolates an oxacillin resistance, while being in almost one-quarter of all isolates resistant to rifampicin. In contrast, *S. aureus* displayed in only 7% of all isolates an oxacillin resistance or a rifampicin resistance. Therefore, an empiric therapy might be sufficient in the case of *S. aureus*, while being insufficient for treatment of coagulase-negative staphylococci. Nevertheless, previous studies have already highlighted the relevance of coagulase-negative staphylococci. Charalambous et al., as well as Wimmer et al., described a poor outcome after PJI with coagulase-negative staphylococci [2,20]. Therefore, vancomycin must be discussed as an antibiotic therapy before identification of the causative pathogen for effective antibiotic treatment, as vancomycin resistance is still rare in Gram-positive staphylococci. An additional advantage of its application is its coverage of *E. faecium*. Although PJI with enterococci is still rare with described rates of 2.3–15% in the literature in comparison to staphylococci, an empiric first-line therapy with beta-lactam-antibiotics might be not the right choice [5]. According to our data, in 19% of the PJIs caused by enterococci, *E. faecium* could be identified as the causative pathogen. For sufficient treatment, application of vancomycin is needed. Thankfully, vancomycin resistance did not play a relevant role, with only 2 *E. faecium* isolates of 57 enterococcus species being resistant.

To date, the role of Gram-negative pathogens in patients with PJI of the hip or knee remains controversial. Indisputably, detection rates of Gram-negative pathogens in PJI are described with 6 to 23%, which is consistent with our results, as 13.1% of the isolates were Gram-negative bacteria [21]. Nevertheless, the taxa of the most frequently detected species often differ. Bjerke-Kroll et al. described *P. aeruginosa* as the most frequently detected (26.7% of all Gram-negative pathogens) [14]. In contrast Zmistowski detected *E. coli* (30.2%) as the most frequently detected Gram-negative pathogen, while describing similar rates for *E. coli* with 25% [14,21]. In our cohort, overall detection rates of *E. coli* were similar (26.2% of all Gram-negative pathogens), although only 8.7% of all Gram-negative bacteria have been identified as *P. aeruginosa*. We were not able to identify a trend for Gram-negative pathogens. Frequencies of detection of different Gram-negative species remained inconsistent. We have not detected all species in all years analysed. Interestingly, *E. coli* was the most frequently detected Gram-negative pathogen, except in 2018 and 2019, where *P. mirabilis* was more frequently detected. We must admit that we cannot explain this change over time.

Antibiotic susceptibility testing revealed high resistance rates of Gram-negative bacteria against ciprofloxacin and piperacillin-tazobactam, which did not change significantly over the course of time. In the literature, Benito et al. reported an increase in resistance of Gram-negative bacteria against ciprofloxacin of up to 18% [22]. The resistance of Gram-negative bacteria against ciprofloxacin remains here decisive, as ciprofloxacin is the cornerstone in treatment of PJI caused by Gram-negative bacteria and, in general, wild-type isolates of Gram-negative bacteria should be susceptible to ciprofloxacin [23]. As for Germany, several official warnings exist to limit the use of ciprofloxacin as a result of possible side effects (tendinitis, tendon rupture, tremor, peripheral neuropathy; published at the end of 2019 and 2020), a possible decrease in resistance based on a more restrictive application might be expected within the next year. Nevertheless, the results of our antibiotic susceptibility testing did not show a sudden decrease of resistance against ciprofloxacin in 2021. As infections with Gram-negative bacteria might not be suspected in the majority of the patients with PJI, application of ciprofloxacin and piperacillin-tazobactam should only be performed in the case of previous detection of Gram-negative pathogens. Despite the overall high resistance rates to piperacillin-tazobactam of up to 26.5%, we regard it as a valid first-line therapy in the case of a suspected infection with Gram-negative pathogens and unavailability of the previous results of the antibiotic susceptibility testing. Meropenem remains, in this context, an initial therapy as a valid alternative in selected cases (patient with PJI and septic shock, absent of other treatment alternatives in the case of known antibiotic susceptibility testing), until targeted therapy is possible as only one isolate displayed a resistance. To avoid an increase in resistant strains, application must always be carefully considered.

Our study has some limitations. As we are not able to access previous microbiological results of foreign laboratories or the exact history of the previously performed antibiotic therapies, there is a collection and selection bias. Moreover, the data were collected at a tertiary endoprosthetic referral center, where patients are often transferred to in case of complications (e.g., persisting PJI or patient-related factors such as multimorbidity). In addition, we did not evaluate our rate of culture-negative PJI.

## 4. Materials and Methods

In this retrospective study, we included all consecutive cases of PJI of the hip or knee joint at a tertiary endoprosthetic referral center in Germany between January 2016 and December 2021 and microbiological detection of causative pathogens in specimens (tissue biopsies, synovial fluid, sonication fluids of explanted prostheses) obtained intraoperatively. The study was approved by our local institutional review board.

Intraoperatively collected tissue specimen (shredded and homogenized) as well as sonication fluid (0.5 mL) were plated on Columbia agar with 5% sheep blood, MacConkey agar, chocolate agar, and Sabouraud agar (Becton & Dickinson, Bergen County, NJ, USA), while 1 mL was pipetted into thioglycollate broth (Becton & Dickinson, Bergen County, NJ, USA). For anaerobic cultures, Schaedler and kanamycin/vancomycin agar plates (Becton & Dickinson, Bergen County, NJ, USA) were struck with 0.5 mL sonication fluid or with shredded and homogenized tissue specimens. All cultures were grown at 5% CO_2_ and 35 °C for at least 14 days. In parallel, sonication fluid was inoculated into PEDS medium blood culture flasks (Becton & Dickinson, Bergen County, NJ, USA) and incubated in a Bactec FX blood culture system (Becton & Dickinson, Bergen County, NJ, USA) for 14 days. Joint aspirates were inoculated in PEDS medium blood culture flasks (Becton & Dickinson, Bergen County, NJ, USA) and incubated in a Bactec FX blood culture system (Becton & Dickinson, Bergen County, NJ, USA) for 14 days. Matrix-assisted LaserDesorption/Ionization Time-Of-Flight Mass Spectrometry (MALDI-TOF MS, (bioMérieux, Nürtingen, Germany)) was used to identify the pathogens. Antimicrobial susceptibility testing was performed with an automated antimicrobial susceptibility testing system, Vitek2 (bioMérieux, Nürtingen, Germany). In the case of detection of anaerobic pathogens, susceptibility testing was carried out with a semiautomated microtiter broth dilution system (MICRONAUT; Merlin, Bornheim, Germany). For interpretation of antimicrobial susceptibility, the EUCAST clinical breakpoints (v. 12.0, 2022) were applied.

A PJI was defined according to Parvizi et al., with fulfilling one of the following criteria: (1) a sinus tract communicating with the prosthesis, (2) isolation of the same microorganism from two or more cultures/tissue biopsies obtained from the infected joint or (3) isolation of one microorganism in the intraoperative cultures with additional evidence of an infection of the inlying implant (positive histology, presence of purulence, elevated serum erythrocyte sedimentation rate, elevated C-reactive protein and elevated synovial white blood cell count) [24].

For better description of the included patients, we recorded patient demographics, weight, site of arthroplasty, surgery time (cutting/suture), comorbidities, performed procedures and preoperative anemia. Based on the time interval between surgery and infection, we classified the infection as the following, using modified criteria according to Izakovicova et al. [19]: acute early onset (occurring within 6 weeks after surgery with symptom duration less than 3 weeks), and persisting early onset (occurring within 6 weeks after surgery with symptom duration more than 3 weeks; e.g., persisting infection after failed treatment of acute PJI), acute late onset (occurring after 6 weeks after surgery with symptom duration less than 3 weeks) and chronic late onset (occurring after 6 weeks after surgery with symptom duration more than 3 weeks). If during this study patients underwent surgery several times of the same joint, only the first episode was recorded.

A polymicrobial PJI was defined as detection of more than one microorganism isolated from the intraoperative tissue biopsies, sonication or synovial fluid. The microbiological profiles of all pathogens were analyzed.

Statistical analysis: Data were collected in Microsoft Excel 2022 (Microsoft Corporation, Redmond, WA, USA). Statistical analysis was carried out with SPSS statistics 28 for Windows (SPSS, Inc., an IBM company, Chicago, IL, USA). Descriptive statistics, including arithmetic mean values and standard deviations, were calculated. Data are given as means ± standard deviation (SD), if not indicated otherwise.

To analyse categorial data, Pearson’s chi-squared test was used to test for an association. A *p*-value less than 0.05 was considered statistically significant. To better analyse trends, we divided the 6-year period into two 3-year intervals (2016–2018 and 2019–2021). In detail, the test was used to assess the trends in distribution of the microorganisms and antibiotic resistance in patients with PJI. In the case of multiple testing, a Bonferroni correction was performed for adjusting the α-value.

## 5. Conclusions

Knowledge of antibiotic susceptibility in causative pathogens in patients with PJI plays an important role in avoiding an insufficient antibiotic therapy in the case of an empiric therapy or an unknown pathogen. As the majority of PJI is caused by Gram-positive pathogens, with coagulase-negative staphylococci being the most frequently identified bacteria, oxacillin resistance must be considered. For the highest antimicrobial coverage in the case of an empiric therapy/unknown pathogen, vancomycin might be chosen. In the case of a suspected infection with Gram-negative bacteria, augmentation with piperacillin-tazobactam should be considered.

## Figures and Tables

**Table 1:** Demographics of hip and knee periprosthetic joint infections.

Demographic Characteristics	2016–2021	2016–2018	2019–2021
Number of patients [*n*]	493	220	273
male	243 (49.2%)	107 (48.6%)	135 (49.5%)
female	250 (50.8%)	113 (51.4%)	138 (50.5%)
PJI of the hip	293 (59.4%)	120 (54.5%)	173 (63.4%)
right	125 (25.3%)	54 (24.5%)	71 (26.0%)
left	168 (34.1%)	66 (30.0%)	102 (37.4 %)
PJI of the knee	200 (40.4%)	100 (45.5%)	100 (36.6%)
right	100 (20.2%)	49 (22.3%)	50 (18.3%)
left	100 (20.2%)	51 (23.2%)	50 (18.3%)
Age (Mean ± SD) [years]	69 ± 11	70 ± 11	69 ± 12
BMI (Mean ± SD) [kg/m^2^]	29.99 ± 8.85	30.06 ± 8.39	29.91 ± 8.73
Preoperative Creatinine [mg/dL]	1.02 ± 0.76	1.08 ± 0.99	0.98 ± 0.485
Preoperative C-reactive Protein [mg/L]			
-acute early onset [*n* = 90]	84.16 ± 68.95	79.81 ± 57.55 [*n* = 42]	89.44 ± 77.86 [*n* = 48]
-persisting early onset [*n* = 4]	85.78 ± 43.54	/	85.78 ± 43.54 [*n* = 4]
-acute late onset [*n* = 90]	131.65 ± 109.04	129.2 ± 102.64 [*n* = 34]	51.40 ± 73.39 [*n* = 56]
-chronic late onset [*n* = 309]	53.56 ± 79.62	55.94 ± 86.50 [*n* = 144]	51.41 ± 73.39 [*n* = 165]
Number of previous surgeries	4.71 ± 4.1	4.67 ± 4.23	4.75 ± 4.34
Comorbidities			
Hypertension	437 (88.6%)	203 (92.1%)	234 (85.7%)
Smoking	147 (29.8%)	66 (30%)	81 (29.6%)
Diabetes mellitus	175 (35.5%)	80 (36.3%)	94 (34.4%)
Alcoholism	51 (10.3%)	25 (11.3%)	26 (9.5%)
Cirrhosis	41 (8.3%)	18 (8.1%)	23 (8.4%)
Malignancy	72 (14.6%)	36 (16.3%)	36 (13.1%)
Rheumatoid arthritis	54 (10.9%)	20 (9.1%)	34 (12.4%)
Immunosuppression	100 (20.3%)	50 (22.7%)	50 (18.3%)
chron. kidney disease	142 (28.8%)	63 (28.6%)	79 (28.9%)

**Table 2:** Culture data over a six-year period by organism.

	2016	2017	2018	2019	2020	2021	Detection in PJI of the Hip	Detection in PJI of the Knee	Total by Species
**Aerobic Gram-positive**	66	74	76	90	88	81	276	199	475 (77.61%)
Coagulase-negative staphylococci	38	36	43	54	56	48	174	101	275 (44.93%)
*S. aureus*	15	11	11	15	20	14	44	42	86 (14.05%)
*E. faecalis*	7	9	11	7	2	10	29	17	46 (7.52%)
*E. faecium*	2	5	0	4	0	0	6	5	11 (1.8%)
*Streptococcus* species	4	11	9	9	7	8	17	31	48 (7.84%)
*Micrococcus*	0	1	1	0	1	1	3	1	4 (0.65%)
*Granulicatella adiacens*	0	0	0	1	0	0	1	0	1 (0.16%)
*Kocuria rhizophila*	0	0	0	0	1	0	1	0	1 (0.16%)
*Corynebacterium* species	0	1	1	0	1	0	1	2	3 (0.49%)
**Rod-shaped or anaerobic Gram-positive**	2	9	1	8	10	7	23	14	37 (0.61%)
*C. acnes*	2	4	1	5	9	6	15	12	27 (4.41%)
*C. avidum*	0	4	0	1	0	1	6	0	6 (0.98%)
*C. tertium*	0	0	0	1	0	0	1	0	1 (0.16%)
*Erysipelothrix rhusiopathiae*	0	1	0	0	0	0	1	0	1 (0.16%)
*Pseudoarthrobacter sulfonivorans*	0	0	0	1	0	0	0	1	1 (0.16%)
*Peptoniphilus coxii*	0	0	0	0	1	0	0	1	1 (0.16%)
**Gram-negative**	6	12	13	14	15	20	52	28	80 (13.07%)
*E. coli*	2	7	2	2	2	6	10	11	21 (3.43%)
*P. mirabilis*	1	1	5	5	1	2	9	6	15 (2.45%)
*P. vulgaris*	1	0	0	0	0	0	1	0	1 (0.16%)
*E. cloacae* complex	1	1	0	1	2	3	8	0	8 (1.31%)
*Serratia marcescens*	0	0	1	2	3	0	5	1	6 (0.98%)
*P. aeruginosa*	0	1	0	2	1	3	6	1	7 (1.14%)
*K. pneumoniae*	1	2	3	1	4	0	5	6	11 (1.8%)
*K. aerugenes*	0	0	1	0	1	2	3	1	4 (0.65%)
*K. oxytoca*	0	0	1	0	0	1	1	1	2 (0.33%)
*Acinetobacter baumannii* complex	0	0	0	1	0	0	1	0	1 (0.16%)
*Bacteroides vulgatus*	0	0	0	0	0	1	1	0	1 (0.16%)
*Citrobacter koseri*	0	0	0	0	0	2	2	0	2 (0.33%)
*Porphyromonas somerae*	0	0	0	0	1	0	0	1	1 (0.16%)
**Fungus** (*Candida* species)	1	3	4	2	3	2	10	5	15 (2.45%)
*Bacillus* species	0	0	1	0	2	1	2	2	4 (0.65%)
*Brevibacterium luteolum*	0	0	1	0	0	0	1	0	1 (0.16%)
Total by year	75	98	96	114	118	111	364	248	612 (100%)

**Table 3:** Antibiotic resistance of selected pathogens against selected antibiotics.

Pathogen		2016		2017		2018		2019		2020		2021		2016–2021		Total
		r	s	r	s	r	s	r	s	r	s	r	s	r	s	
*S. aureus*	oxacillin	3	11	0	11	1	10	1	14	0	20	1	13	6	79	85 ^a^
	rifampicin	3	11	1	10	1	10	0	15	0	20	1	13	6	79	85 ^a^
	vancomycin	0	14	0	11	0	11	0	15	0	20	0	14	0	85	85 ^a^
coagulase negative staphylococci	oxacillin	24	13	22	14	33	10	26	27	34	19	22	25	161	108	269 ^b^
	rifampicin	8	30	11	25	11	32	12	41	19	36	6	42	67	206	273 ^c^
	vancomycin	0	38	1	35	0	43	1	53	0	55	0	47	2	271	271 ^d^
*Streptococcus* species	penicillin G	0	4	0	11	0	9	0	9	0	7	0	8	0	48	48
*Enterococcus* species	ampicillin	2 ^f^	7	5 ^f^	9	0	11	4 ^f^	7	0	2	0	10	11	46	57 ^a^
	vancomycin	0	9	1 ^f^	13	0	11	1 ^f^	10	0	2	0	10	2	54	56 ^e^
Gram-negative bacteria	piperacillin-tazobactam	3	3	0	12	1	12	4	9	8	6	8	12	24	54	78 ^e^
	ciprofloxacin	0	6	3	9	1	12	5	9	3	11	6	13	18	60	78 ^e^
	meropenem	0	6	0	12	0	13	1	13	0	14	0	20	1	78	79 ^a^

r = resistant; s = susceptible; ^a^: susceptibility testing according to EUCAST for one isolate not possible; ^b^: susceptibility testing according to EUCAST for six isolates not possible; ^c^: susceptibility testing according to EUCAST for two isolates not possible; ^d^: susceptibility testing according to EUCAST for four isolates not possible. ^e^: susceptibility testing according to EUCAST for two isolates not possible; ^f^: only *E. faecium*.

## Data Availability

The data presented in this study are available in the manuscript.
